# Evaluation of Satisfaction Levels Following Orthognathic Treatment in Adult Patients: A Systematic Review

**DOI:** 10.7759/cureus.73846

**Published:** 2024-11-17

**Authors:** Abdulmalek M.H. Almasri, Mohammad Y. Hajeer, Kinda Sultan, Ossama Aljabban, Ahmad Salim Zakaria, Jacqueline B. Alhaffar

**Affiliations:** 1 Department of Orthodontics, Faculty of Dentistry, University of Damascus, Damascus, SYR; 2 Department of Endodontics and Restorative Dentistry, Faculty of Dentistry, University of Damascus, Damascus, SYR; 3 Department of Orthodontics, School of Dental Sciences, Universiti Sains Malaysia, Kota Bharu, MYS; 4 Department of Oral and Maxillofacial Surgery, Faculty of Dentistry, University of Damascus, Damascus, SYR

**Keywords:** conventional orthognathic surgery, orthodontic treatment, patient perception of improvement, patient-reported outcome, patient-reported outcome measure, patient satisfaction, satisfaction, skeletal class ii deformities, skeletal class iii deformities, surgical intervention

## Abstract

Dentofacial deformities can significantly impact an individual's quality of life, affecting facial aesthetics, self-esteem, and overall well-being. The combined orthognathic surgery-orthodontic treatment is the preferred approach for correcting moderate-to-severe deformities. However, patient satisfaction following orthognathic surgery remains a crucial outcome measure, influenced by various factors, including the type of malocclusion, surgical procedure, and demographic characteristics. This systematic review aimed to synthesize the available evidence regarding patient satisfaction following orthognathic surgery, exploring the effects of the type of malocclusion, surgical procedure, age, and gender on satisfaction rates, addressing a gap left by previous outdated reviews. A comprehensive literature search was conducted across multiple databases, including PubMed®, Scopus®, Web of Science™, and Embase®. Eligibility criteria were defined using the PICOS (population, intervention, comparison, outcomes, and study design) framework. Cochrane’s ROBINS-I (Risk of Bias In Non-randomized Studies-of Interventions) tool was employed for non-randomized intervention studies within clinical controlled trials to assess the risk of bias. In parallel, a revised version of the Newcastle-Ottawa scale determined the methodological quality of cohort and cross-sectional studies. Sixteen studies were analyzed, revealing satisfaction levels ranging from 83% to 100%. Findings indicate that class III malocclusion patients report higher satisfaction than class II patients and satisfaction varies based on surgical type, with bimaxillary procedures generally yielding better outcomes. While most studies found no significant correlation between satisfaction and demographic factors such as age and gender, some suggested younger patients may express higher satisfaction and that female patients might report lower satisfaction levels. The review highlights the importance of effective patient communication and expectation management in achieving optimal satisfaction outcomes in orthognathic surgery. Limitations such as memory bias and methodological diversity across studies restrict the ability to perform meta-analyses, underscoring the need for further research in this area.

## Introduction and background

Dentofacial deformities arise from imbalances among the facial and dental bone structures, which develop at different rates and can affect facial aesthetics and the harmony of the stomatognathic system [1]. Skeletal deformities may lead to malocclusion and neuromuscular imbalances, affecting essential functions such as respiration, mastication, and phonation. Research indicates adverse impacts on self-esteem, self-confidence, and mental health [2]. When paired with orthodontic treatment, orthognathic surgery is the optimal method for correcting moderate-to-severe deformities [3]. This procedure realigns and repositions the maxilla concerning the skull base, effectively treating malocclusion, particularly in patients with dentofacial deformities [4]. Surgical correction can range from adjusting groups of teeth to completely repositioning the mandible and maxilla, aiming for functional occlusion, facial symmetry, and healthy orofacial structures [5]. In addition to functional issues, dentofacial deformities affect psychosocial well-being and overall quality of life [6]. Research has investigated the psychological, social, physical, functional, and aesthetic impacts of orthognathic surgery before and after the procedure [7].

Orthognathic surgery involves various procedures to correct dental, skeletal, and facial discrepancies, aiming to improve musculoskeletal function and overall quality of life [8]. A multidisciplinary approach is essential, including orthodontists, maxillofacial surgeons, nurses, dieticians, and sometimes psychiatrists. Orthognathic surgery is usually paired with orthodontic fixed appliances to address malocclusion linked to dentofacial deformities, aiming to achieve facial harmony and enhance aesthetics [9].

Patient satisfaction is a key outcome shaped by pre-surgery expectations and the information provided by the medical team [10]. Despite generally high satisfaction rates following orthognathic surgery, some patients express dissatisfaction despite successful outcomes [11]. Patient satisfaction following orthognathic surgery is usually assessed using questionnaires. These questionnaires are typically administered at various time points during the postoperative period. Some studies evaluate satisfaction one month after the surgical procedure [12], while others assess it at three to six months post-surgery [13], years after the operation [14-16], or upon completion of post-surgical orthodontics [17]. Reports indicate high rates of patient satisfaction following combined orthodontic and surgical interventions. Those who have undergone orthognathic treatment have experienced numerous psychological benefits, such as boosted self-esteem and heightened self-confidence [18]. In contrast, dissatisfaction can also occur due to patients’ unachieved expectations [11]. Understanding patients’ expectations and views is crucial to achieving patient satisfaction and success in orthognathic treatment.

A review of the published literature revealed variations in satisfaction levels following orthognathic surgery. Alkharafi et al. [19] reported that 96.4% of participants felt no regret undergoing combined orthodontic and surgical treatments. On the other hand, satisfaction with the orthognathic surgery result was reported by 87% in Finlay et al.'s study [20]. Al-Asfour et al. [21] investigated patient satisfaction across different surgical interventions, finding satisfaction levels of 95.8% for Le Fort I osteotomy, 94% for bilateral sagittal split osteotomy (BSSO), and 90.9% for bimaxillary jaw surgery.

Two earlier systematic reviews on this topic have been published [22,23]. Both reviews are now outdated. The first, published in 2016, concluded eight studies [23]. Of these, five had a high risk of bias, and three used non-validated questionnaires. The other systematic review was published in 2017 [22], and the review authors admit that they might have missed potential eligible studies because they had only determined three bibliographic databases (PubMed, Embase, PsycINFO). This relatively out-of-date review justifies the need to explore and analyze any emerging new evidence in this field. Hence, this systematic review consolidated the evidence on patient satisfaction levels following orthognathic treatment. The focused review question of this report was "What are the levels of patient satisfaction following orthognathic surgery?".

## Review

Materials and methods

Scoping Search

Before finalizing the systematic review procedure, a PubMed scoping search was performed to verify the existence of prior systematic reviews and identify potentially relevant publications. This review followed the guidelines established by the Preferred Reporting Items for Systematic Reviews and Meta-Analyses (PRISMA) [24].

Review Eligibility Criteria

The search strategy used the PICOS (participants, intervention, comparison, outcomes, and study design) framework. The study participants included in this review were healthy individuals of both genders, aged 17 or older, with malocclusion associated with dentofacial deformities who underwent orthognathic surgery. The intervention group should include any orthognathic treatments with a presurgical orthodontic phase. In comparative studies, the comparison group should consist of patients treated with conventional or untreated orthodontic treatment. The outcome measures under assessment were patient satisfaction after orthognathic treatment measured by a visual analog scale (VAS), numerical rating scale (NRS), verbal rating scale (VRS), the dental impact of daily living questionnaire (DIDL), post-surgical patient satisfaction questionnaire (PSPSQ), oral health impact profile questionnaire (OHIP), orthognathic quality of life questionnaire (OQLQ), or any other validated patient satisfaction questionnaire. This review encompasses a variety of study designs, including randomized controlled trials (RCTs), non-RCTs (CCTs), cohort studies, and cross-sectional studies. No limitations were imposed on the publication dates or language of the studies included.

Information Sources

A comprehensive electronic literature search was conducted utilizing various databases, including PubMed®, Scopus®, Web of Science™, the Cochrane Central Register of Controlled Trials, Google™ Scholar, Embase®, Trip, and OpenGrey. References in the included papers were manually reviewed to identify any additional relevant research that might have been missed during the computerized searches. The review process involved an electronic examination of ClinicalTrials.gov alongside the World Health Organization’s International Clinical Trials Registry Platform to locate clinical trials that are currently ongoing, have been completed, and are published.

Search Strategy and Study Selection

Table 3 (Appendix) includes a compilation of the keywords used in the search strategy. Table 4 (Appendix) contains comprehensive details regarding the electronic search strategy employed. The eligibility of the selected articles was determined through a two-phase process. During the first phase, two reviewers (AMHA and MYH) independently examined the titles and abstracts of satisfaction with orthognathic treatment identified through all electronic databases. In the subsequent phase, the reviewers thoroughly evaluated the full-text articles to establish their final eligibility. In instances of disagreement, a third review author (KS) intervened to render a decision as needed.

Data Collection Process

Two reviewers (AMHA and MYH) extracted data from the included studies and organized them into tables. In instances of disagreement, the third author (ASZ) was tasked with mediating the issue until a consensus was reached. The tables include the following comprehensive details: the authors' names, study context, and publication year, along with methods covering study design, questionnaire type, and timing. Additionally, they provide detailed participant demographics, including sample size, age, gender, and type of surgery.

Assessing the Risk of Bias of the Included Studies

Initially, the risk of bias for the included articles was determined by the two reviewers (AMM and MYH) separately using Cochrane’s risk of bias in non-randomized studies of intervention (ROBINS-I) for clinical controlled trials CCTs [25], and the modified version of the Newcastle-Ottawa scale for cohort and cross-sectional studies [26]. Following this, the assessments made by the two reviewers were compared. In cases where their opinions diverged, a third reviewer (KS) was engaged to reach a consensus. The seven domains of the ROBINS-I tool for CCTs were assessed and categorized as having low, moderate, critical, no information, or serious risk of bias. Subsequently, the overall risk of bias for each study was evaluated using the following criteria: low risk if all fields were assessed as low risk; moderate risk if all fields were assessed as low or moderate risk; serious risk if one or more fields were assessed as having a serious risk, but none were assessed as critical; critical risk if one or more fields were assessed as critical; and no information if there was a lack of information in one or more key bias categories without clear indication of serious or critical risk. Cohort and cross-sectional research were the intended uses for the modified Newcastle-Ottawa scale. This evaluation tool is structured around eight domains and grouped into three key categories to analyze the studies: patient selection, comparison of study groups, and outcome assessment. A rating system was utilized to evaluate the quality of the studies. Research that met high-quality standards with minimal bias could receive a maximum of 9 stars. Studies rated at 8, 7, or 6 stars were classified as moderate quality, whereas those rated at 5 stars or fewer were considered lower quality.

The Quality of the Evidence

Two reviewers (AMM and MYH) independently assessed the quality of the evidence for each outcome. Their evaluations were then compared. When disagreements persisted despite discussions, a third reviewer (ASZ) was consulted to make a final decision.

Results

Literature Search Flow and the Retrieved Studies

**Figure 1:**
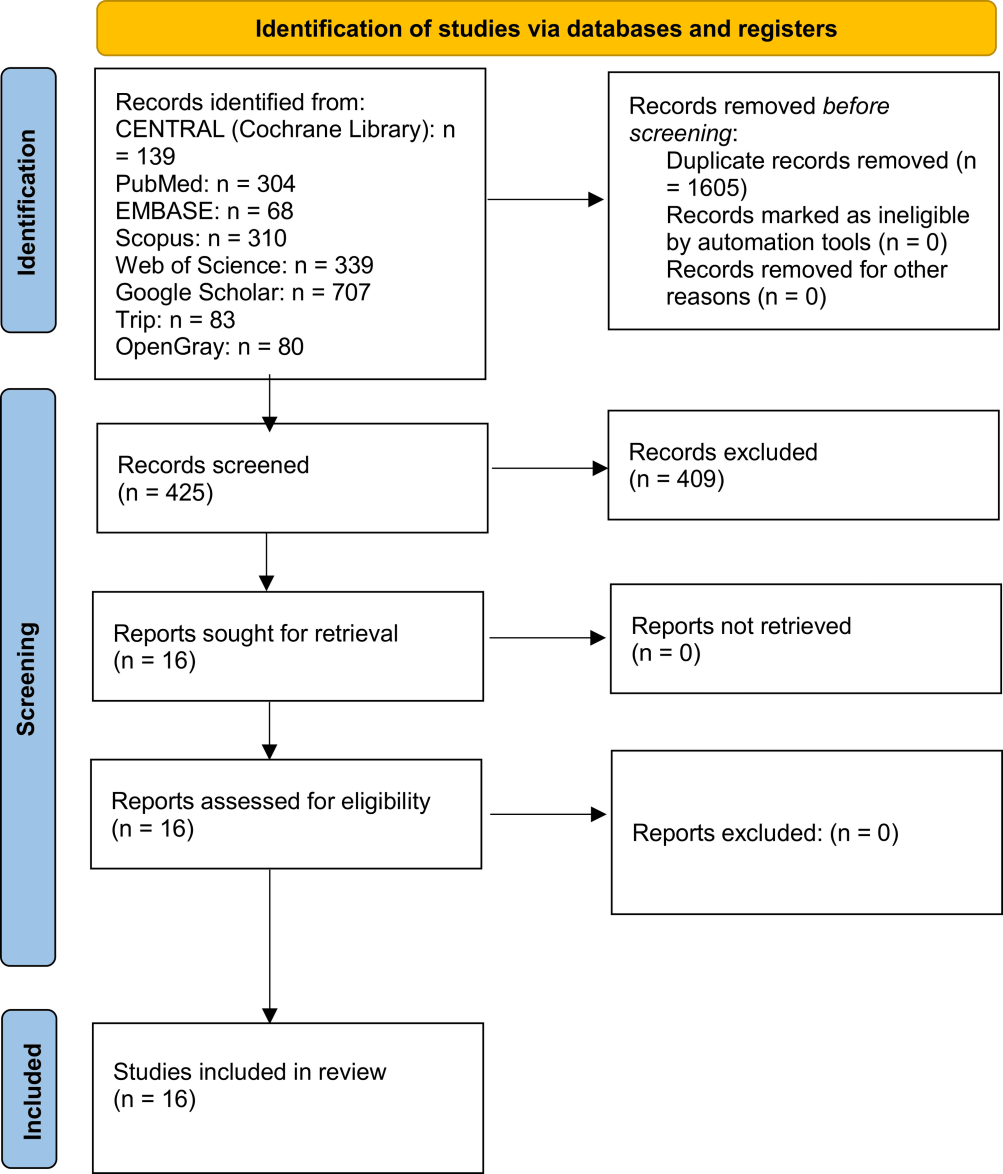
The Preferred Reporting Items for Systematic Reviews and Meta-Analyses (PRISMA) flow diagram of study identification, screening, and inclusion in the review.

The systematic electronic search across diverse databases and reference lists generated 2,030 references. After removing duplicate entries, 425 citations were subjected to an in-depth review. Title and abstract screening excluded 409 documents, leaving 16 full-text records for further eligibility assessment. Ultimately, the systematic review included 16 studies [14,15,19,21,27-38]. Figure 1 illustrates the PRISMA flow chart detailing the inclusion and selection processes.

Characteristics of the Included Studies

Table 1 contains the features of the included studies. Out of these studies, one was a CCT [32], two were cohort studies [28,37], and the other thirteen studies had a cross-sectional design [14,15,19,21,27,29-31,33-36,38]. All of them were presented in English. These studies were carried out across ten countries, including Germany [29,34,35], Finland [15,30], Norway [14,36], the USA [31,33], Denmark [32], Kuwait [19,21], the UK [27], Brazil [37], Oman [28], and Turkey [38].

**Table 1:** Characteristics of the included studies in this systematic review. Exp.: experimental group, BSSO: bilateral sagittal split osteotomy, PSPSQ: post-surgical patient satisfaction questionnaire, CCT: clinical controlled trial, VAS: visual analog scale, OQLQ: orthognathic quality of life questionnaire. * in years

Author, Year, and Country	Study design	Number of patients (M/F)	Mean age*	Type of malocclusion	Type of surgery	Timing of satisfaction assessment	Satisfaction assessment tool
Bock et al., 2007, Germany [29]	Cross-sectional	102 (35/67)	24.3 ± 5.3	Mandibular prognathism: 48%, Mandibular retrognathism: 32%, Open bite: 13%, Laterognathism: 7%	BSSO: 51%, Le Fort-I osteotomy: 11%, Bimaxillary osteotomies: 21%, Miscellaneous: 17%	11-141 months after surgery Mean: 47 months	Non-validated questionnaire Closed-form questions (yes or no answer)
Pahkala and Kellokoski, 2007, Finland [15]	Cross-sectional	82 (29/53)	32 ± 10.2	Mandibular retrognathism: 78%, Mandibular prognathism: 22%	BSSO	1.8 ± 0.5 years after surgery	Non-validated 3-items questionnaire
Espeland et al., 2008, Norway [14]	Cross-sectional	516 (235/281)	27.2 ± 10.3	Skeletal class I: 15%, Skeletal class II: 29%, Skeletal class II: 56%	BSSO: 55%, Le Fort-I osteotomy: 16%, Bimaxillary osteotomies: 19%, Other procedure: 10%	3 years after surgery	Non-validated author-devised questionnaire 7-items graded on a 4-point Likert scale
Posnick et al., 2008, USA [33]	Cross-sectional	42 (15/27)	25 ± 10.3	Vertical maxillary excess: 40%, Mandibular deficiency: 21%, Other: 39%	Le Fort-I osteotomy: 31%, Bimaxillary osteotomies: 69%	After fixed appliance debonding and at least 6 months after surgery	PSPSQ graded on a 7-point Likert scale
Oland et al., 2010, Denmark [17]	CCT	Exp.: 118 (51/67); Control: 47 (18/29)	Exp.: 28.8 ± 8.2; Control: 31.5 ± 8.8	Not reported	BSSO: 15%, Le Fort-I osteotomy: 48%, Bimaxillary osteotomies: 37%	After post-surgical orthodontics was completed (at least 12 months)	PSPSQ graded on a 5-point Likert scale
Rustemeyer et al., 2010, Germany [34]	Cross-sectional	77 (40/37)	23.4 ± 4.9	Skeletal class III	Bimaxillary osteotomies (Le Fort-I and BSSO)	13.2 ± 2.1 months after surgery	Non-validated questionnaire 6-items graded on an 11-point VAS 7-items closed-form (yes or no answer) 1 open-ended question
Alkharafi et al., 2014, Kuwait [19]	Cross-sectional	74 (22/52)	21.1 ± 4.1	Not reported	BSSO: 30%, Le Fort-I osteotomy: 8%, Bimaxillary osteotomies: 62%	Between 6 months and 10 years after post-surgical orthodontics was completed	Pilot-tested with three patients’ questionnaire 9-items graded on a 3-point Likert scale
Kufta et al., 2016, USA [31]	Cross-sectional	37 (16/21)	23.5 ± 10.9	Not reported	BSSO: 32%, Le Fort-I osteotomy: 41%, Bimaxillary osteotomies: 16%, Bimaxillary osteotomies with genioplasty: 11%	6-12 months after surgery	16 items graded on a 6-point Likert scale questionnaire
Al-Asfour et al., 2018, Kuwait [21]	Cross-sectional	66 (24/42)	25.1 ± 3.9	Not reported	BSSO: 8% Le Fort-I osteotomy: 9%, Bimaxillary osteotomies: 83%	6 months to 7 years after surgery	OQLQ graded on VAS
Al-Hadi et al., 2018, UK [27]	Cross-sectional	118		Not reported	BSSO: 24%, BSSO and genioplasty: 6%, Le Fort-I osteotomy: 15%, Bimaxillary osteotomies: 55%	23.84 ± 15.7 months after surgery	Questionnaire graded on 5-point Likert scale
Torgersbråten et al., 2021, Norway [36]	Cross-sectional	60 (13/47)	25.5	Skeletal class II	BSSO: 30%, Le Fort-I osteotomy: 33%, Bimaxillary osteotomies: 37%	3 years after surgery	5-item non-validated author-devised questionnaire
Thiem et al., 2021, Germany [35]	Cross-sectional	119 (53/66)	31.3 ± 11.1	Skeletal class II: 63%, Skeletal class III: 33% Posterior crossbite: 2.5%, Open bite: 1.5%	BSSO: 44%, Le Fort-I osteotomy: 4%, Bimaxillary osteotomies: 52%	59 ± 19.7 months after surgery	18 items non-validated author-devised questionnaire graded on the Likert scale
Kamaraian et al., 2021, Finland [30]	Cross-sectional	57 (19/38)	49 ± 10.2	Skeletal class II: 78%, Skeletal class III: 22%	BSSO	10-15 years after surgery	12 items questionnaire graded on VAS
Vicente et al., 2023, Brazil [37]	Cohort	25 (10/15)	28.6	Skeletal class III	BSSO: 24%, Le Fort-I osteotomy: 40%, Bimaxillary osteotomies: 36%	6 months after surgery	22 items OQLQ
Alsenaidi et al., 2024, Oman [28]	Cohort	136 (51/85)	25.1 ± 6.5	Not reported	BSSO: 17%, Le Fort-I osteotomy: 18%, Bimaxillary osteotomies: 30%, Bimaxillary osteotomies and genioplasty: 19%, Genioplasty: 16%	T0: 2 weeks after surgery T1: 3 months after surgery T2: 6 months after surgery	Modified OQLQ graded on VAS
Yazici et al., 2024, Turkey [38]	Cross-sectional	73 (25/48)	18-21 years: 18%, 22-30 years: 65%, 31-65 years: 17%	Skeletal class II: 40%, Skeletal class III: 60%	BSSO: 17.8%, Le Fort-I osteotomy: 13.7%, Bimaxillary osteotomies: 65.7%, Genioplasty: 2.8%	6-12 months after surgery	Validated questionnaire graded on 7-point Likert scale

A total of 1,749 participants were included in these 16 studies (1,034 females and 715 males). These studies were published between 2007 and 2024, and all engaged patients of both genders. No studies targeted a single gender. The findings demonstrated significant variability in sample sizes, ranging from 25 to 516 patients and ages from 17 to 65 years.

Several studies include patients with skeletal class II and class III deformities, irrespective of the origin of the dentofacial deformity (i.e., the upper or lower jaw) [30,38]. Two studies focus on mandibular retrognathism or prognathism [15,29], while another addressed patients with vertical maxillary excess and mandibular retrognathism [33]. Two papers addressed exclusively to class III malocclusion [34,37], whereas others concentrated on class II malocclusion [36]. Additionally, one study includes a range of deformities such as class II, class III, anterior open bite, and posterior crossbite [35]. However, six studies did not specify the type of skeletal malocclusion in their patient demographics [19,21,27,28,31,32,35].

Regarding the surgical procedure performed, 14 out of 16 studies included a mixture of surgical procedures [14,19,21,27-29,31-38], and two studies focused exclusively on a single surgical procedure (BSSO) [15,30]. The BSSO procedure was used in all included studies, whereas the Le-Fort I procedure was employed in 13 studies [14,28,30-32,34-38]. Bimaxillary osteotomy was the procedure conducted in 14 studies [14,19,21,27-29,31-38], whereas genioplasty was used in only four studies [27,28,31,38].

The authors utilized various questionnaires to evaluate patient satisfaction. Two studies employed a post-surgical patient satisfaction questionnaire (PSPSQ) [32,33], while three studies used the orthognathic quality of life questionnaire (OQLQ) [21,28,37]. Additionally, five studies implemented validated questionnaires graded on a Likert scale [19,27,30,31,38], while the other six studies relied on non-validated questionnaires [14,15,29,34-36]. Additionally, there were variations in the timing of questionnaire administration across the studies. Some studies administered questionnaires within 6-12 months after surgery [31-34,37,38], others between one and three years [14,15,27,36], and some had a broader range, fluctuating between six months and 15 years [19,21,28-30,35].

Risk of Bias and Quality of the Included Studies

**Figure 2:**
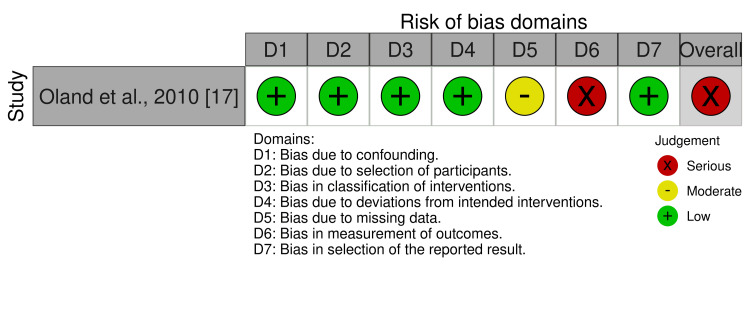
Risk of bias of this included study according to the seven domains of the employed tool.

As shown in Figure 2, the only included CCT was classified as having a serious risk of bias due to bias in the measurement of the outcome because the outcome assessors knew which intervention each study participant received. More details about the risk of bias evaluation of the included CCT are given in Table 5 (Appendix).

The Newcastle-Ottawa scale was employed to evaluate the methodological quality scores of the remaining 15 cohort and cross-sectional studies, as detailed in Table 2. Two studies reached the maximum of nine stars and were classified as having a high level of quality, while eight studies were considered to have a moderate level of quality, and five studies were judged to have a low level of quality. In selecting the study groups, two studies were awarded five stars, six received four stars, and seven scored three stars or less. Regarding the evaluation of the outcome of interest, five studies were rated three stars, six received two stars, and the remaining four were assigned one star.

**Table 2:** Quality assessment for cohort and cross-sectional studies using the modified Newcastle-Ottawa scale. Studies considered high quality and at low risk of bias can receive a maximum of 9 stars; articles achieving 8, 7, and 6 stars have moderate quality; and articles with 5 stars or fewer indicate low quality.

Study	Selection (****)				Comparability (**)	Outcome (***)		Total score
	Representativeness of the sample	Sample size	Non-respondents	Ascertainment of exposure	The subjects in different outcome groups are comparable, based on the study design or analysis. Confounding factors are controlled	Assessment of the outcome	Statistical test	
Bock et al., 2007 [29]	*	*	-	*	-	*	*	5
Pahkala and Kellokoski, 2007 [15]	*	*	*	*	*	*	-	6
Espeland et al., 2008 [14]	*	*	*	**	*	**	*	9
Posnick et al., 2008 [33]	-	*	*	*	-	*	*	5
Rustemeyer et al., 2010 [34]	*	*	*	*	*	*	*	7
Alkharafi et al., 2014 [19]	*	*	-	*	*	*	*	6
Kufta et al., 2016 [31]	-	*	*	*	*	-	*	5
Al-Asfour et al., 2018 [21]	*	*	*	*	*	**	*	8
Al-Hadi et al., 2018 [27]	*	-	*	*	*	*	*	6
Torgersbråten et al., 2021 [36]	*	*	*	**	*	**	*	9
Thiem et al., 2021 [35]	*	*	*	*	*	**	*	8
Kamaraian et al., 2021 [30]	*	*	*	*	-	-	*	5
Vicente et al., 2023 [37]	-	-	*	*	*	*	-	4
Alsenaidi et al., 2024 [28]	*	*	*	*	*	**	*	8
Yazici et al., 2024 [38]	-	*	*	*	*	*	*	6

Main Findings

Patient satisfaction levels following orthognathic surgery: Despite the variations between the included studies, patient satisfaction after orthognathic surgery was generally high, ranging from 83% in the Torgersbråten et al. survey [36] to 100% in Pahkala et al.'s survey [15]. Most articles used different questionnaires at different assessment times to assess satisfaction with orthognathic treatment, and the extracted data (sample size, patient ages, male-female ratio, type of malocclusion, type of surgery) were not homogenous enough to perform a meta-analysis.

Effect of the type of malocclusion on patient satisfaction: Six studies explored patient satisfaction following orthognathic surgery for class II malocclusion [14,15,29,30,36,38]. In a survey conducted by Bock et al. [29], patient satisfaction was evaluated 11-141 months post-surgery, revealing a satisfaction rate of 78.7% among patients with class II mandibular retrognathism. Similarly, studies by Torgersbråten et al. [36] and Espeland et al. [14] reported satisfaction rates of 83% three years after surgery.

Satisfaction rates for class III patients were generally higher than those of class II patients. Most studies found a high satisfaction rate of more than 90% [34,38], and 100% in the Kufta et al. study [31]. Only one study by Bock et al. reported a relatively low satisfaction rate of 79.5% [29].

Effect of surgery type on patient satisfaction: Out of the 16 included studies, eight addressed the correlation between the type of surgery and satisfaction rate [14,15,21,29,30,32,34,36]. For BSSO procedures, satisfaction rates varied significantly. The Torgersbråten et al. survey reported a satisfaction rate of 72.2% [36], while the studies by Kamaraian et al. and Pahkala et al. achieved a perfect satisfaction rate of 100% [15,30]. Other notable findings include satisfaction rates of 77% in both the Bock et al. and Øland et al. studies [29,32] and higher rates of 92% and 94% in the Espeland et al. and Al-Asfour et al. studies, respectively [14,21]. For Le-Fort I procedures, patient satisfaction rates also varied widely. The Bock et al. survey reported a satisfaction rate of 72.7% [29], while the Al-Asfour et al. survey recorded a high of 95.8% [21]. The Øland et al. study found a satisfaction rate of 78.6% [32], and the Espeland et al. study reported 89.7% [14]. Additionally, the Torgersbråten et al. survey indicated a satisfaction rate of 90% [36]. When it comes to bimaxillary procedures, satisfaction rates were somewhat closer. The Torgersbråten et al. survey reported a satisfaction rate of 86.4% [36], while the Al-Asfour et al. study found a rate of 91% [21]. The Espeland et al. study reported a satisfaction rate of 92% [14], and both the Oland et al. and Pahkala and Kellokoski studies recorded a rate of 93% [15,32].

Effect of age and gender on patient satisfaction: Of the 16 included articles, 11 studies explored the correlation between patient satisfaction and age or gender [14,15,21,29-32,34,36-38]. Eight studies found no significant association between patient satisfaction and age or gender [15,21,29-32,34,36]. However, Espeland et al. discovered that, among the 8% of dissatisfied patients, 79% were female [14]. Similarly, Vicente et al. identified a statistically significant difference between genders, indicating that female patients were less satisfied six months post-surgery (p < 0.05) [37]. Regarding age, only one paper found a correlation between age and satisfaction. Yazici et al. found that younger patients were more satisfied and reported that 60% of satisfied patients were in their 20s [38].

Discussion

Risk of Bias of the Included Studies

In our systematic review, we assessed the risk of bias across the included studies to evaluate the reliability of our findings. Since most studies on patient satisfaction following orthognathic surgery are retrospective, the reliability of their findings is questionable. These findings underscore the heterogeneity in the methodological quality of the included studies. The serious risk of bias in the CCT and the varying quality levels in the cohort and cross-sectional studies emphasize the need to interpret the review's results carefully. Future research should aim to enhance methodological rigor and minimize bias to provide more reliable and generalizable evidence.

Patient Satisfaction Levels Following Orthognathic Surgery

There was a notable agreement across the studies, highlighting that patients generally expressed high satisfaction levels after receiving orthognathic treatment. This agreement can be attributed to several factors. First, the significant aesthetic improvements resulting from the surgery often lead to enhanced facial harmony and attractiveness, which, in turn, greatly boost patients’ self-confidence and self-esteem [15,34]. Second, the surgery addresses functional issues such as malocclusions, improving bite, chewing, speaking, and even breathing, which are critical for daily comfort and overall quality of life [21,28]. The quality of care provided and positive interactions with the orthodontic-surgical team are crucial to patient satisfaction [31]. However, it is paramount to have clear and effective communication about the realistic outcomes of the treatment to align patients’ expectations with achievable results, ensuring their overall satisfaction [27].

Effect of the Type of Malocclusion on Patient Satisfaction

Six studies addressed class II patient satisfaction following orthognathic surgery [14,15,29,30,36,38]; satisfaction rates ranged from 78.7% in Bock et al. cross-sectional study [29] to 83% in Torgersbråten et al. and Espeland et al. studies [14,36]. Regarding class III patients’ higher satisfaction rates ranged from 93% in the Rustemeyer et al. [34] study to 100% in the Kamaraian et al. study [30]. This difference in satisfaction rate can be explained by the fact that class III surgical correction leads to more noticeable and dramatic improvements in facial aesthetics, and the patients may experience more pronounced functional improvements, such as better bite alignment and improved chewing efficiency, which contribute to their overall satisfaction [38]. The psychological impact of these improvements can also be more substantial for class III patients, as they often face more severe functional and aesthetic challenges pre-treatment [34].

Effect of Surgery Type on Patient Satisfaction

Significant differences were noted between studies in patient satisfaction scores ​​after orthognathic surgery, whether the surgical procedure was BSSO or Le-Fort I. The BSSO procedure satisfaction rate ranged from 72.2% in the Torgersbråten et al. survey [36] to 100% in studies by Kamaraian et al. [30] and Pahkala et al. [15], while it ranged from 72.7% in Bock et al. survey [29] to 95.8% in Al-Asfour et al. study [21] for the Le-Fort I procedure. After perusing the materials and methods of each study individually, no clear reason for these differences was found; maybe patient satisfaction is related to the type of malocclusion rather than the type of surgical procedure.

Effect of Age and Gender on Patient Satisfaction

Among the 11 studies that addressed the correlation between age or gender and patient satisfaction [14,15,21,29-32,34,36-38], eight studies found no significant correlation [15,21,29-32,34,36]. In contrast, Espeland et al. [14] and Vicente et al. [37] found that female patients were less satisfied with the final result, and this can be explained by the fact that female patients may experience higher levels of anxiety and stress related to surgical procedures and recovery [14]. They might also be more sensitive to postoperative discomfort and complications. Additionally, the psychological impact of changes to facial aesthetics might be more pronounced for women, leading to a more critical evaluation of the results [37]. Yazici et al. found a correlation between age and patient satisfaction in their study [38]. They reported that younger patients tended to be more satisfied with their orthognathic treatment. This can be attributed to the fact that younger individuals are often at a stage where their social and professional lives are just beginning, making a facial appearance that conforms to societal norms particularly important to them [38].

Limitations of the Current Systematic Review

A clear limitation of this review is the presence of memory bias, deriving from the varying times at which satisfaction was assessed across the included studies. The timing could significantly influence the perception of satisfaction. Additionally, the method of data collection - whether face-to-face interviews, mailed questionnaires, or structured phone interviews - could result in different interpretations of the outcomes. Other notable limitations include the inability to perform a meta-analysis due to substantial variations among the included studies, such as differences in the type of malocclusion, the type of surgery performed, and patient demographics.

## Conclusions

Patient satisfaction following orthognathic surgery remains high despite the methodological variations across studies. Satisfaction rates range from 83% to 100%, with class III patients reporting higher satisfaction levels than class II patients. The type of surgery also impacts satisfaction, with bimaxillary procedures often yield higher satisfaction rates than single-jaw surgeries. While most studies found no significant correlation between satisfaction and demographic factors such as age and gender, others highlighted that younger patients are more satisfied, and females may report lower satisfaction rates. These findings underscore the need for personalized patient care and clear communication to manage expectations and optimize outcomes in orthognathic surgery.

## Appendices

**Table 3:** Keywords used in the search strategy.

Components of the search strategy	Relevant keywords
Orthognathic	Orthognathic Surgery, Orthognathic Surgical Procedures, Orthognathic Surgeries, Orthognathic Surgery, Orthognathic, Maxillofacial Orthognathic Surgery, Jaw Surgery, Maxillofacial Surgeries.
Satisfaction	Satisfaction, Patient Preference, post-surgical patient satisfaction questionnaire, PSPSQ, Dental Impact of Daily Living, DIDL, Quality of Life, PROMs, Patient-reported Outcome Measures, Satisfaction, Likert Scale, Patient-oriented outcome measures.
Type of surgery	Le Fort, Osteotomy, Mandibular Osteotomy, Maxillary Osteotomy, Bilateral Sagittal Split Osteotomy, BSSO, intraoral vertical ramus osteotomy, IVRO, bimaxillary osteotomies.

**Table 4:** Electronic search strategy in different bibliographic databases.

Database	Search Strategy
CENTRAL	#1 orthognathic surgery OR orthognathic surgical procedures OR orthognathic surgeries OR orthognathic OR maxillofacial orthognathic surgery OR jaw surgery OR maxillofacial surgeries OR maxillofacial orthognathic OR Le Fort OR osteotomy OR mandibular osteotomy OR maxillary osteotomy OR BSSO OR bilateral sagittal split osteotomy. #2 satisfaction OR patient satisfaction OR patient preference OR patient-reported outcome measures OR PROMs OR dental impact of daily living OR DIDL OR post-surgical patient satisfaction questionnaire OR satisfaction Likert scale. #3 #1 OR #2 #4 #1 AND #2
EMBASE	#1 orthognathic surgery OR orthognathic surgical procedures OR orthognathic surgeries OR orthognathic OR maxillofacial orthognathic surgery OR jaw surgery OR maxillofacial surgeries OR maxillofacial orthognathic OR Le Fort OR osteotomy OR mandibular osteotomy OR maxillary osteotomy OR BSSO OR bilateral sagittal split osteotomy. #2 satisfaction OR patient satisfaction OR patient preference OR patient-reported outcome measures OR PROMs OR dental impact of daily living OR DIDL OR post-surgical patient satisfaction questionnaire OR satisfaction Likert scale. #3 #1 OR #2 #4 #1 AND #2
PubMed	#1 orthognathic surgery OR orthognathic surgical procedures OR orthognathic surgeries OR orthognathic OR maxillofacial orthognathic surgery OR jaw surgery OR maxillofacial surgeries OR maxillofacial orthognathic OR Le Fort OR osteotomy OR mandibular osteotomy OR maxillary osteotomy OR BSSO OR bilateral sagittal split osteotomy. #2 satisfaction OR patient satisfaction OR patient preference OR patient-reported outcome measures OR PROMs OR dental impact of daily living OR DIDL OR post-surgical patient satisfaction questionnaire OR satisfaction Likert scale. #3 #1 OR #2 #4 #1 AND #2
Scopus	#1TITLE-ABS-KEY (orthognathic surgery OR orthognathic surgical procedures OR orthognathic surgeries OR orthognathic OR maxillofacial orthognathic surgery OR jaw surgery OR maxillofacial surgeries OR maxillofacial orthognathic OR Le Fort OR osteotomy OR mandibular osteotomy OR maxillary osteotomy OR BSSO OR bilateral sagittal split osteotomy). #2 TITLE-ABS-KEY (satisfaction OR patient satisfaction OR patient preference OR patient-reported outcome measures OR PROMs OR dental impact of daily living OR DIDL OR post-surgical patient satisfaction questionnaire OR satisfaction Likert scale). #3 #1 OR #2 #4 #1 AND #2
Web of Science	#1TS= (orthognathic surgery OR orthognathic surgical procedures OR orthognathic surgeries OR orthognathic OR maxillofacial orthognathic surgery OR jaw surgery OR maxillofacial surgeries OR maxillofacial orthognathic OR Le Fort OR osteotomy OR mandibular osteotomy OR maxillary osteotomy OR BSSO OR bilateral sagittal split osteotomy). #2TS= (satisfaction OR patient satisfaction OR patient preference OR patient-reported outcome measures OR PROMs OR dental impact of daily living OR DIDL OR post-surgical patient satisfaction questionnaire OR satisfaction Likert scale). #3 #1 OR #2 #4 #1 AND #2
Google Scholar	#1 (orthognathic surgery OR orthognathic surgical procedures OR orthognathic surgeries OR orthognathic OR maxillofacial orthognathic surgery OR jaw surgery OR maxillofacial surgeries OR maxillofacial orthognathic OR Le Fort OR osteotomy OR mandibular osteotomy OR maxillary osteotomy OR BSSO OR bilateral sagittal split osteotomy) AND (satisfaction OR patient satisfaction OR patient preference OR patient-reported outcome measures OR PROMs OR dental impact of daily living OR DIDL OR post-surgical patient satisfaction questionnaire OR satisfaction Likert scale).
Trip	(orthognathic surgery OR orthognathic surgical procedures OR orthognathic surgeries OR orthognathic OR maxillofacial orthognathic surgery OR jaw surgery OR maxillofacial surgeries OR maxillofacial orthognathic OR Le Fort OR osteotomy OR mandibular osteotomy OR maxillary osteotomy OR BSSO OR bilateral sagittal split osteotomy) AND (satisfaction OR patient satisfaction OR patient preference OR patient-reported outcome measures OR PROMs OR dental impact of daily living OR DIDL OR post-surgical patient satisfaction questionnaire OR satisfaction Likert scale).
OpenGrey	#1 Orthognathic and satisfaction

**Table 5:** Risk of bias judgments of the included controlled clinical trial.

Study	Bias due to confounding	Bias in the selection of the participants	Bias in the classification of intervention	Bias due to deviations from intended interventions	Bias due to missing data	Bias in the measurement of outcome	Bias in the selection of the reported results	Overall
Oland et al., 2010 [17]	Low risk: no confounding factors detected	Low risk: All participants who have been eligible for the target trial were included in the study	Low risk: Intervention status is well-defined	Low risk: No deviations from intended interventions were detected	Moderate risk: outcome data were not available for all participants. Missing data were not related to the intervention	Serious risk: No information about outcome assessors blinding and the results may be influenced by knowledge of the intervention received by study participants	Low risk: pre-defined outcomes mentioned in the methods section seemed to have been reported	Serious risk
